# Genetic diversity of rotavirus infection among young children with diarrhoea in the Kassena-Nankana Districts of Northern Ghana: a seasonal cross-sectional survey

**DOI:** 10.11604/pamj.2023.44.148.36783

**Published:** 2023-03-28

**Authors:** Flavia Kaduni Bawa, Mohamed Mutocheluh, Sylvester Donne Dassah, Patrick Ansah, Abraham Rexford Oduro

**Affiliations:** 1 Department of Clinical Microbiology, Kwame Nkrumah University of Science and Technology, Kumasi, Ghana,; 2 Navrongo Health Research Center, Research and Development Division, Ghana Health Service, P.O. Box 114, Navrongo, Ghana,; 3 Department of Biochemistry and Forensic Sciences, C.K Tedam University of Technology and Applied Sciences, Navrongo, Ghana

**Keywords:** Rotavirus, diarrhoea, vaccines, genotypes, Northern Ghana, children, parasites, prevalence

## Abstract

**Introduction:**

diarrhoea disease is a global health concern, persisting as one of the top five causes of morbidity and mortality in children. Viral aetiology of childhood diarrhoea is often associated with rotavirus infection of which preventable vaccines exist. Here we document circulating strains of rotavirus in the Kassena-Nankana Districts of Northern Ghana nearly a decade after the introduction of the rotavirus vaccine.

**Methods:**

a cross-sectional survey of children aged 0-60 months was conducted in six health facilities within the Kassena-Nankana Districts. Faecal samples obtained from the children were analysed and characterized for rotavirus detection and genotyping using Semi-Nested Polymerase Chain Reaction.

**Results:**

a total of 263 stool samples were analyzed. Out of which 14.8% and 18.6% of the diarrhoea cases were of rotavirus and parasitic etiologies respectively, with 17.4% being co-infections. Almost 27.5% of rotavirus diarrhoeal cases resulted in hospitalization. Household size (p=0.035), location (p=0.018), treatment outcome (p=0.007), vomiting (p=0.039), season (p=0.017) and month of sampling (p=0.000) were significantly associated with rotavirus infection. The rotavirus genotypes identified were G1P8, G3P6, G4P9, G10P6 and G12P8. Rotavirus vaccine-type, G1P8 was absent in Kassena-Nankana West District.

**Conclusion:**

the prevalence of rotavirus was low compared to the pre-vaccination era. Also, a new rotavirus strain, G4P9 was identified to be circulating in the study area which calls for surveillance measures and more studies to better understand the situation for appropriate public health intervention.

## Introduction

Diarrhoea is one of the top five causes of morbidity and mortality for children less than five years of age [1]. According to the World Health Organization (WHO), the burden of childhood diarrhoeal disease has been estimated at 1.7 billion per annum globally. Even though this is seen as an improvement from decades ago when cases were about 4 billion per annum, this disease remains on top of the charts, accounting for the death of about 525,000 children yearly [2]. According to the Lancet´s Global Burden of Disease (GBD) in 2020, 90% of diarrhoea deaths occurred in South Asia and sub-Saharan Africa [3]. Diarrhoea in general has a wide range of infectious causes such as viral, bacterial, and protozoan. Viruses such as rotaviruses, noroviruses, adenoviruses, caliciviruses, astroviruses, and enteroviruses are responsible for about 70% of diarrhoeal diseases worldwide with rotaviruses and noroviruses being the most common cause [4]. Vaccines now exist against rotavirus which alone accounts for 90% of the viral aetiology of diarrhoea. The common human genotypes of rotavirus circulating globally are the G1P [8], G2P [4], G3 [P8], G4 [P8] and G9 [P8] strains with new strains emerging from developing countries [5]. Rotarix, a two-dose regimen vaccine targeted at the G1P [8] and non-G1 genotypes and Rotateq, a three-dose regimen vaccine targeted at the G1, G2, G3, G4 and P [8] genotypes of the rotavirus are vaccines being used in several countries worldwide [6,7]. In April 2012, Ghana introduced the use of Rotarix for infants at 4 and 10 weeks of age [8] which has been in use till July 2019 when a switch was made to the Rotavac vaccine [9].

Diarrhoeal disease has remained one of the causes of hospitalization and death in Ghana for several years [10]. A recent study shows that hospitalization due to rotavirus has significantly declined from 49.7% to 27.8% after the introduction of the Rotarix vaccine [11]. A separate study in urban Ghana reported a rotavirus prevalence of 58% before the implementation of the vaccine [12]. Two years following the vaccine's implementation in Kumasi, rotavirus was still found to account for 20.7% of acute childhood diarrhoea, the highest compared to other microbial pathogens such as bacteria and parasites [13]. Rotavirus is known to exhibit wide strain diversity with the emergence of new strains being common [14]. A study by Binka *et al*. found several non-typeable strains that have been speculated to be a result of a shift in primer binding sites or emerging new strains [12]. It was also found that only 41% of the circulating strains in Ghana contain G types 1-4 compared to 95% in developed countries [15]. There are currently no surveillance measures in place to track changing circulating strains in the population. This poses a high risk of genotype replacement by non-vaccine-type genotypes. This calls for surveillance measures and more studies to better understand the situation for appropriate intervention measures to be put in place. Here we report rotavirus genotypes associated with childhood diarrhoea in the Kassena-Nankana Districts of Northern Ghana, a decade since the introduction of the Rotarix vaccine.

## Methods

### Study site

The study was conducted in the Navrongo Health Research Centre (NHRC) using the Navrongo Health and Demographic Surveillance System (NHDSS). This system monitors the health and demographic parameters of the two districts where the study took place [16]. Data collection took place at the Kassena-Nankana East Municipal and West Districts often referred to as the Kassena-Nankana Districts (KNDs). The KNDs is located approximately between latitude 10°30' and 11°00' North, longitude 1°00' and 1°30' West and covers about 1,674 square kilometres of Sahelian savannah with a population of about 170, 000. The main occupations of the inhabitants of the KNDs are subsistence farming and the rearing of ruminants. Although rainfall in this area is variable, the average annual rainfall in the district is about 850 mm and occurs almost entirely between June and October. There is one major dam, the Tono irrigation dam, and several smaller dams and dugouts dotted around the study area, which provide water for limited vegetable cultivation and animals during the long dry season. The area is predominantly rural, and the district health directorates carry out immunization exercises at static clinics, outreaches in the communities and house-to-house immunizations through home visits and supplementary immunization activities. There are Community Health and Planning Services (CHPS) units in each community to facilitate access and delivery of primary health care in the area. Participants for this study were identified from the War Memorial Hospital, Paga District Hospital, Navrongo Health Centre, Wisdom Star Clinic, Kologo Health Centre and Biu Health Centre.

### Study design and population

**Figure 1:**
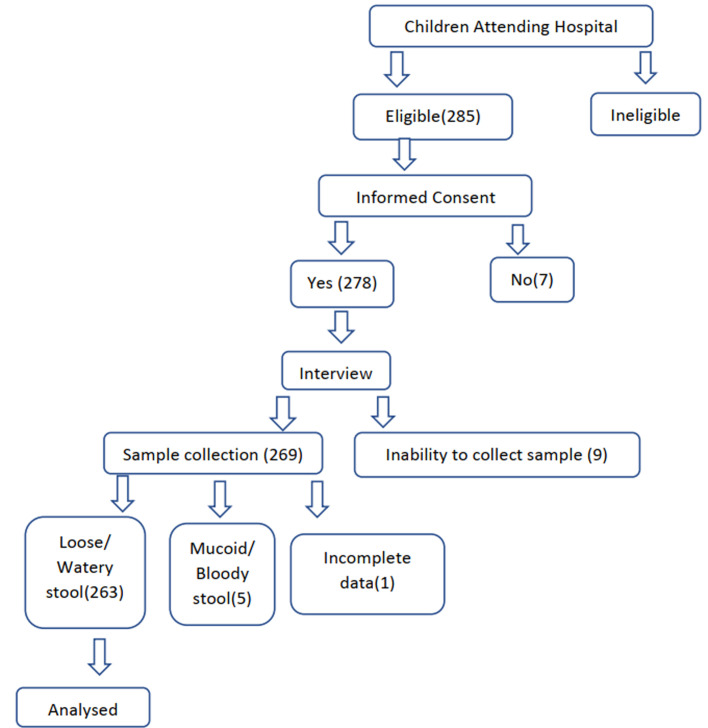
participants' recruitment procedure and enrolment criteria flow chart

This study was a hospital-based cross-sectional study of children zero to sixty months presenting with moderate to severe diarrhoea. Participants' recruitment procedure and enrolment criteria are shown in Figure 1. Children were enrolled after informed consent was obtained from their parent/guardian. This study was conducted between September 2020 and March 2021 during both the dry and wet seasons. Children below the age of five reporting to the health facilities with watery diarrhoea and living within the health demographic system of NHRC were included in the study. Those who did not meet these criteria were excluded. A pre-tested semi-structured questionnaire was used to collect sociodemographic and clinical data. All data forms were checked for completeness and any omissions were corrected before being entered into a pre-designed excel data form.

### Specimen collection and processing

About a spoonful of stool was collected directly into a sterile, wide-mouth container with a lid. For participants who were unable to produce stool, a rectal swab was performed using a sterile cotton swab. These swabs were then placed inside a solution of Phosphate Buffered Saline (PBS) to preserve the sample. Stool samples were labelled with a unique participant´s identification number and transported on ice immediately to the Navrongo Health Research Center Laboratory for analysis. Stool samples collected were first described in terms of colour, presence of blood or mucous (for exclusion from the study), and whether the stool was formed, semi-formed, watery or loose. Stool samples were also examined physically for the presence of adult worms. Wet mounts were prepared for stool samples and examined for the presence of parasites/protozoa upon arrival at the laboratory. The remaining stool samples were processed using PBS and stored in a -20°C freezer till analysis. The swab sticks with their storage medium were frozen as well till further analysis. Information on the presence or absence of malaria parasites was obtained from routine laboratory examinations at the hospital if requested by the clinician. A portion of each stool sample was suspended in normal saline to help view the motility of trophozoites as well as other stages of development of the parasites. A drop of the suspension was placed on a clean, sterile slide and covered with a coverslip. Slides were observed under 10x and 40x objective lenses for motile trophozoites and larvae, eggs, and cysts of parasites.

### RNA extraction and semi-Nested PCR

Frozen stool samples were brought out of the freezer and made to thaw at room temperature before use. The RNA extraction procedure was performed using a nucleic acid extraction kit (Guangzhou LBP Medicine Science and Technology Co Ltd; China) according to the manufacturer's instructions. A two-step semi-nested PCR procedure was used to determine rotavirus presence in samples and genotypes. First-round VP7 amplifications were performed using Qiagen OneStep RT-PCR Kit (Cat No. 210212). Thin-walled tubes (0.2ml) were labelled for each sample including a positive and negative control. A PCR master mix was prepared and placed on ice. This master mix was made up of 10μl of 5x Qiagen OneStep RT-PCR buffer, 2μl of 10mM dNTP mix, 2μl Qiagen One-Step RT-PCR enzyme mix, and 25μl of RNase-free water for each reaction. For VP7 amplification, 3μl of each 20pmol Beg9 and End9 were added to all sample tubes. Con2 and Con3 were used for VP4 amplification. Five microliters (5μl) of the extracted RNA were added to the primers in each reaction tube. The samples were denatured for 5 minutes at 94°C and transferred immediately to an ice bath. Thirty-nine (39μl) of the PCR master mix was added to each tube and mixed. The tubes containing the samples were placed in a thermocycler (Bio-Rad, USA) and incubated using the following cycles conditions: reverse transcription at 50°C for 30 minutes, initial PCR activation at 95°C for 15 minutes, both for one cycle each, then denaturation at 94°C for 30s, 60°C for 30s, 72°C for 1 min all for 30 cycles, and 72°C for 10 min for one cycle.

The concentration of DNA was checked using a Qubit Fluorometer 4.0. These concentrations ranged from 0.644ng/μl to 4.28ng/μl. One percent (1%) agarose gel was prepared with 5μl of SafeView classic dye. Samples with dye and a 1kb ladder were loaded and run at 90v for 45 minutes and were visualized under a gel documentation system (BioRad, United States of America). Images of the gel were interpreted to determine the status of each sample. The second amplification was performed using the nested PCR protocol of the Qiagen one-step RT-PCR kit. A second amplification involved using RVG9 and type-specific primers (aAT8, aBT1, aCT2, aDT4, aET3, aFT9, G10 and G12) for VP7. Con3 and type-specific primers (1T-1, 2T-1, 3T-1, 4T-1, and 5T-1) were used for VP4 second amplification. A genotype master mix was prepared to contain 0.5μl each of these primers at 100pmol concentration, 1μl of dNTPs, 5μl of Qiagen OneStep RT-PCR buffer, 1μl of Qiagen One-Step RT-PCR enzyme mix, and 12.5μl of RNase free water in a 25μl reaction. Twenty-four microliters (24μl) of the mix were added to the labelled tubes. One microliter (1µl) of positive first-round RT-PCR products was added to each of the tubes containing the master mix. The tubes were placed in a thermocycler and activated at 95°C for 15 minutes then run for 25 cycles at 94°C for 30s, 60°C for 2 minutes, 72°C for 1 minute, and one cycle at 72°C for 10 minutes. The PCR fragments were run on 2% TAE agarose gel at 95 volts for 45 minutes with a 100bp molecular marker to determine the genotype of the rotavirus strain.

### Statistical analysis

Data was entered and analysed using IBM SPSS Version 21. All descriptive statistics and analyses were done using the same software. Simple proportions were presented using bar graphs and pie charts. Graphs and tables were generated using Microsoft Excel. Bivariate and multiple logistic regressions were used to establish the association between rotavirus infection and potential risk factors. Results were presented in confidence intervals (95% CI), odds ratios (OR) and P-values were significant if less than 0.05.

### Ethics approval and consent to participate

Scientific and ethical clearance was obtained from the Kwame Nkrumah University of Science and Technology (KNUST) Committee on Human Research Publication and Ethics (CHRPE/AP/355/20) as well as from the Navrongo Health Research Centre Institutional Review Board (NHRCIRB385) before the commencement of the study. Permission was obtained from the Upper East Regional Health Directorate and all heads of institutions where samples were collected. Informed consent was obtained from the guardians of children before enrolment procedures were initiated. Samples and case report forms were coded to anonymize study participants.

## Results

### Background characteristics of study participants

A total of 278 participants consented and were recruited into the study from two hospitals and four health centres. Fifteen participants were excluded from the analysis for non-completeness of data. Eventually, 263 samples were included in the final analysis. Out of these 263 participants data analyzed, 53.6% (141) were males while the females were 46.4% (122). The average age for females was 17.62 months and that of males was 17.27 months, with an overall average of 17.46 months (Table 1). During the period of the rainy season from September 2020 to mid-October 2020, 56 (21.3%) participants with diarrhoea were recruited while 207 (78.7%) were recruited from November 2020 to early March 2021 in the dry season (Table 1). Most of the cases (34.6%) were recruited from the Navrongo Health Centre. About 13% (34/263) of the participants recruited were on admission at the time of enrolment.

**Table 1:** demographic characteristics of participants

Variable	Categories	Frequency (263)
Age (Months)	< 11	96(36.6)
	12-23	107(40.8)
	24-35	38(14.5)
	36-60	22(8.0)
Gender	Males	141(53.6)
	Females	122(46.4)
Marital Status	Married	225(86.2)
	Unmarried	36(13.8)
Occupation	Salaried worker	29(11.1)
	Self-Employed	133(51.0)
	Unemployed	99(37.9)
Education	Educated	242(92.7)
	Uneducated	19(7.3)
Type of Feeding	Exclusive	172(65.5)
	Non-Exclusive	91(34.5)
Household size	£5 People	103(39.5)
	>5 People	158(60.5)
Availability of a toilet facility	Yes	195(74.3)
	No	68(25.7)
Facilities of sample collection	War Memorial Hospital	70(26.6)
	Paga District Hospital	15(5.7)
	Navrongo Health Center	91(34.6)
	Wisdom Star Clinic	71(27.0)
	Biu Health Centre	13(4.9)
	Kologo Health Center	30(1.1)
Season of Year	Dry	207 (78.7)
	Wet	56(21.3)
Month of Sample Collection	September	21(8)
	October	39(14.8)
	November	59(22.4)
	December	42(16)
	January	18(6.8)
	February	70(26.6)
	March	14(5.3)
Source of stool specimen	Stool	97(36.9)
	Rectal swab	166(63.1)
Location of Participant	Urban	121(46.4)
	Rural	140(53.6)

### Rotavirus prevalence and associated risk factors

Out of the 263 children screened, 39 (14.8%) were infected with rotavirus. Children aged 21 - 30 months were the most prevalent (25.0% prevalence rate) whereas there was no case recorded in older children (i.e., 51 - 60 months age). During the dry season, the detection among urban-dwelling % while 3.6% of the cases detected were observed during the rainy season. A total of 27.5% of the rotavirus cases resulted in hospital admission while 12.6% were treated and discharged at the outpatient department. The odds of rotavirus case detection among urban-dwelling children were 2.5 higher (95%CI: 1.18 - 5.43, p = 0.017) compared to those from rural area with diarrhoea (Table 2). Small household size was observed to be associated with reduced odds of rotavirus infections compared to larger households (odd ratio, OR adjusted = 0.39 ((95%CI: 0.16 - 0.93), p = 0.034).

**Table 2:** risk factors associated with rotavirus infection

Variable	Categories	Adjusted OR (95%CI)	p-Value
**Age (Months)**	< 10	0.90 (0.596-1.33)	0.615
	11-20		
	21-30		
	31-40		
	41-50		
	51-60		
**Gender**	Males	1.00 (0.48-2.11)	0.993
	Females		
**Location of Participant**	Urban	2.53 (1.18-5.43)	**0.017**
	Rural		
**Household size**	£5 People	0.39 (0.16-0.93)	**0.034**
	>5 People		
**Season of year**	Dry	0.89 (0.14-5.54)	0.897
	Wet		
**Month**	September	0.65 (0.48-0.88)	**0.005**
	October		
	November		
	December		
	January		
	February		
	March		
**Outcome of Treatment**	Discharged	2.53 (1.18-5.43)	**0.017**
	Admitted		

OR: Odds Ratio; CI: Confidence Interval

### Rotavirus genotypes

**Figure 2:**
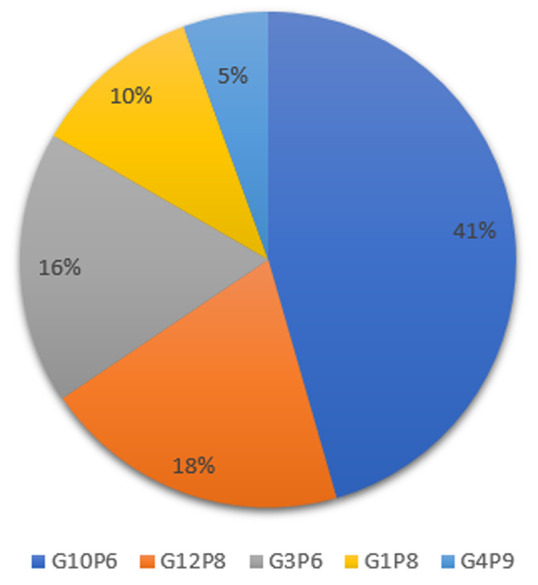
circulating rotavirus genotypes

After first-round amplification, 39 samples were positive for the VP7 gene. Out of this, 35 (89.7%) had VP7 specificities. Four samples did not yield any bands after the second-round amplification hence could not be typed. G10 genotype was the predominant genotype detected with a frequency of 41.3%. This was followed by G12 genotype with 18.0%, G3, G1 and G4 were 15.4%, 10.3%, and 5.1% respectively. All 39 samples were subjected to VP4 amplification and 35 (89.7%) were amplified. All these were VP4 genotyped after second-round amplification. The P6 genotype of the VP4 gene was the predominant genotype which was 62.9% of all VP4 positive samples typed. There was also 31.4% of P8 genotype detected and 5.7% of the rare human VP4 genotype, P9 detected in the samples. In terms of genotype combinations, four of the common rotavirus genotype combinations; G10P6 (41%), G12P8 (18%), G3P6 (16%) and G1P8(10%) (Figure 2) were observed. Also detected was the rare G4P9 (5%) combination.

### Geographic Information Hotspots (GIS) of rotavirus infection

**Figure 3:**
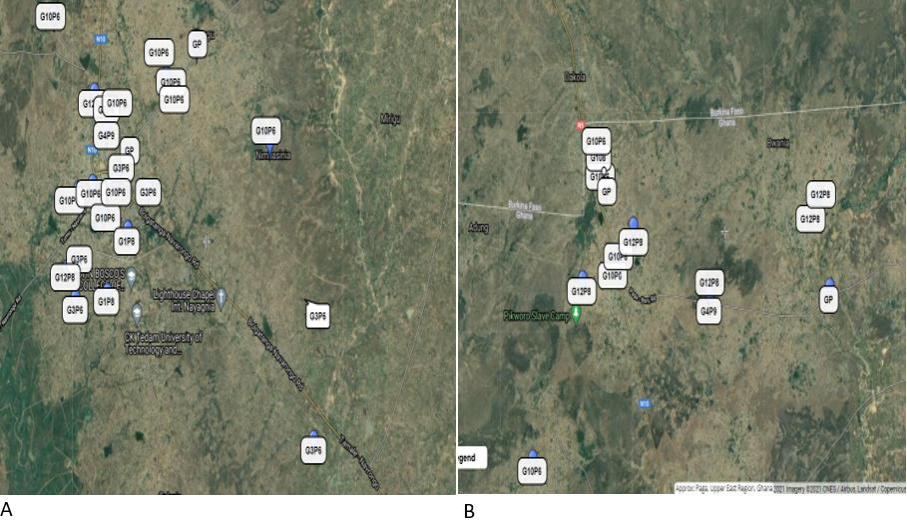
distribution of rotavirus genotypes in the study area: A) distribution of rotavirus genotypes in the Kasena-Nankana West District, B) distribution of rotavirus genotype in the Kasena-Nankana East Municipality

Geospatial distribution of the rotavirus cases shows that majority of the positive cases were from the main towns of the two districts (Paga and Navrongo) (Figure 3 A,B). Hotspots of rotavirus infections in these towns (Paga and Navrongo) were also localized to specific suburbs, Kakungu and Gware in Paga and Nogsenia and Namolo in Navrongo being the most affected. All five different combinations of rotavirus genotypes were detected in the Kassena-Nankana East Municipal (Figure 3B). In the Kassena-Nankana West District, only three different rotavirus genotype combinations (G10P6, G12P8, and G4P9) were detected (Figure 3A), making the former more diverse in rotavirus distribution. The rare G4P9 genotype detected was common in both districts.

### Parasitic aetiology of diarrhoea observed

**Figure 4:**
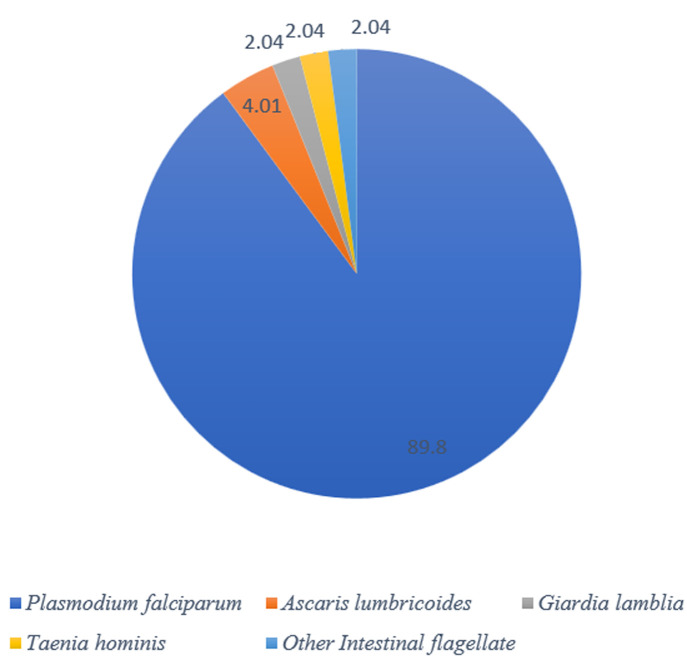
distribution of parasitic cause of diarrhoea

Parasites detected during the study included *Plasmodium falciparum, Ascaris lumbricoides, Trichomonas hominis*, and *Giardia lamblia* (Figure 4). These accounted for 18.63% of all the diarrhoea cases screened. The blood parasite, *P. falciparum* was the most detected parasite. During the rainy season, 23.2% of the diarrhoeal case had parasitic aetiology while during the dry season, it accounted for 17.4% of the infections. Concurrent infection of both rotavirus and parasite was detected in 17.39% of the total cases screed.

## Discussion

A few years before the Rotarix vaccine was introduced in Ghana, the prevalence of rotavirus related diarrhoea among children was 58% [12]. A comparatively low prevalence of 27.8% was found by Akuffu *et al*. in 2017, five years after the vaccine introduction, [17] culminating in an overall decline in the burden of gastroenteritis [11,18]. Out of the 263 participants screened in the present study, 39 (14.8%) were infected with rotavirus. This suggests a further decline considering the nearly 28% prevalence reported in previous studies [13,19,20]. Rotarix vaccine coverage has improved over the years which might account for the prevalence being reported in the present study [10]. There has also been improved hygiene due to the recent protocols on COVID-19 and safe drinking water in the study areas which could also contribute to the low prevalence [21]. The gene-specific analysis of the VP4 and VP7 genes revealed five different combinations. These include G10P6, G12P8, G3P6, G1P8 and G4P9 genotypes. These are among the common rotavirus strains circulating globally with the emergence of the G12P8 genotype seen in several recent studies [22,23]. In Ghana, Armah *et al*. detected G1P8 and G3P6 combinations and the emergence of the G10P6 before the vaccines were introduced [24]. Post-vaccination analysis of rotavirus strains in Ghana by Lartey *et al*. (2018) detected G12P8, G10P6, G3P6 and G1P8 strains as the most predominant genotypes circulating in Ghana [25]. The proportions of these types however differ from what was seen in this study. The smaller proportions of G1P8 detected in the present study suggest the success of the vaccine which is a monovalent vaccine specific to this genotype [26]. These differences in proportions of the other genotypes are likely to be a result of genotype domination by some types over others as time pass.

It was observed that the Kassena-Nankana East Municipal showed great diversity of rotavirus genotypes compared to those in the Kassena-Nankana West District. This was unexpected because the two are adjourning districts. Kassena-Nankana East Municipal is however more populated and developed, accounting for its great diversity in strains detected. A rare rotavirus genotype, the G4P9 genotype was detected in both districts suggesting both areas are at risk of new and emerging animal rotavirus genotypes since both areas have similar animal-rearing characteristics. The time or month of the year sample was collected, was a significant determinant of rotavirus positivity (Table 1). This observation in the seasonality of rotavirus infection was previously reported in the same study area [15]. Children in urban areas were seen to be the most at risk of rotavirus infection compared to those in rural areas (Figure 3). This observation is in contrast to what was reported in Niger, where children from rural communities were most affected [27]. The differences could be due to the lifestyle of the inhabitants of the urban areas in our setting such as attending gatherings with children and sending children to school at an early age which exposes them and makes them more at risk of getting infected. Household size was a significant contributing factor to rotavirus infection corroborating with previous studies where household contacts were considered to greatly impact the burden of disease in children [28]. Although age and gender did not show any statistical significance in causing rotavirus diarrhoea in this study, the age group of 24-35 months reported the highest number of cases. This could be a result of the reduced vaccine efficacy of the rotavirus vaccine in the second year of life [29] making them more vulnerable to rotavirus infections.

Parasitic cause of diarrhoea was shown to be a major concern responsible for 18.6% of the diarrhoea cases screened. This is consistent with findings from a study in neighbouring Burkina Faso which reported that 24.08% of diarrhoea cases tested were of parasitic aetiology [30]. Of the parasites detected in the present study, *Plasmodium falciparum* alone accounted for 89.8% of the infections. The rest were *A. lumbricoides, Giardia Lamblia*, and *T. hominis*. It was not surprising that the malaria parasite was the predominant diarrhoea-causing parasite since it has always been the cause of most children reporting at the hospital [29]. Malaria parasite and the presence of *G. lamblia*, were reported previously in a similar study in Northern Ghana [31]. In this study, 17% of the diarrhoea cases were due to co-infection of rotavirus and parasites. This increases the severity of the infection as it was observed that the odds of rotavirus infection were 2.5 times higher for children admitted with diarrhoea than those seen at the outpatient department.

### Limitations

The major challenge faced during this study was the inability to collect the samples from all the major health centres within the study area which greatly affected the overall prevalence. Another challenge was that children were only brought in after some days of illness. Due to the self-limiting nature of the virus, this could have accounted for the low prevalence found. Also, most parents reported to the health facilities without the vaccination cards of the children, making it difficult to obtain the number of vaccinated children.

## Conclusion

A relatively low prevalence rate of rotavirus infection, 14.8% was observed which suggests the success of various interventions especially the rotavirus vaccine, Rotarix, in eliminating rotavirus infections. The vaccine-type genotype, G1P8 was detected in only one of the districts. The predominant genotype was the G10P6 genotype and a rare animal-type combination, G4P9 was also detected. Factors such as the location of the participant, household size, the outcome of treatment, and season were risk factors for rotavirus infection in the area. Molecular surveillance is recommended to detect vaccine escape mutants, serotype replacement and zoonotic potential.

### What is known about this topic

- *Rotavirus is the leading cause of childhood diarrhoea;*
- *Rotavirus vaccines have played a role in reducing the incidence of childhood diarrhoea;*
- *Genotypes of rotavirus keep changing with time*.

### What this study adds

- *Vaccine strains of rotavirus are eliminated in some populations;*
- *Urban populations are more at risk of rotavirus infections than rural populations;*
- *Parasites play a key role in childhood diarrhoea*.
